# Italian survey on non-intubated thoracic surgery: results from INFINITY group

**DOI:** 10.1186/s12871-021-01514-3

**Published:** 2022-01-03

**Authors:** Giulio Luca Rosboch, Federica Giunta, Edoardo Ceraolo, Federico Piccioni, Francesco Guerrera, Eleonora Balzani, Alessandro Pardolesi, Paolo Albino Ferrari, Davide Tosi, Marco Rispoli, Giudo Di Gregorio, Ruggero Massimo Corso, Roberto Crisci

**Affiliations:** 1 Department of Anesthesia and Intensive Care, AOU Città della Salute e della Scienza, Torino, Italy; 2 Department of Surgical Science, Anesthesia and Intensive Care Unit, University of Turin, Via Verdi, 8 -, 10124 Torino, Italy; 3 Department of Critical and Supportive Care, Fondazione IRCCS Istituto Nazionale dei Tumori, Milan, Italy; 4 Department of Surgical Sciences, Thoracic Surgery Unit, University of Turin, Torino, Italy; 5 Division of Thoracic Surgery, Foundation IRCCS National Cancer Institute of Milan, Milan, Italy; 6 Division of Thoracic Surgery, A. Businco Cancer Center, Azienda Ospedaliera Brotzu, Cagliari, Italy; 7 Division of Thoracic Surgery, Fondazione IRCCS Ca’ Granda Ospedale Maggiore Policlinico, Milan, Italy; 8 Unit of Anestesiology and Intensive Care, Vincenzo Monaldi Hospital, Naples, Italy; 9 Unit of Anestesiology and Intensive Care, ULSS6 Euganea Ospedale di Cittadella (PD), Padova, Italy; 10 Department of Surgery, Anesthesia and Intensive Care Section, “G.B. Morgagni-Pierantoni” Hospital, Forlì, Italy; 11 Thoracic Surgery, University of L’Aquila, L’Aquila, Italy

**Keywords:** Non-intubated thoracic surgery, Video-assisted thoracic surgery, Survey

## Abstract

**Background:**

Non-Intubated Thoracic Surgery (NITS) is becoming increasingly adopted all over the world. Although it is mainly used for pleural operations,, non-intubated parenchymal lung surgery has been less frequently reported. Recently, NITS utilization seems to be increased also in Italy, albeit there are no multi-center studies confirming this finding. The objective of this survey is to assess quantitatively and qualitatively the performance of NITS in Italy.

**Methods:**

In 2018 a web-based national survey on Non-Intubated management including both thoracic surgeons and anesthesiologists was carried out. Reference centers have been asked to answer 32 questions. Replies were collected from June 26 to November 31, 2019.

**Results:**

We raised feedbacks from 95% (55/58) of Italian centers. Seventy-eight percent of the respondents perform NITS but only 38% of them used this strategy for parenchymal surgery. These procedures are more frequently carried out in patients with severe comorbidities and/or with poor lung function. Several issues as obesity, previous non-invasive ventilation and/or oxygen therapy are considered contraindications to NITS. The regional anesthesia technique most used to provide intra- and postoperative analgesia was the paravertebral block (37%). Conversion to general anesthesia is not anecdotal (31% of answerers). More than half of the centers believed that NITS may reduce postoperative intensive care unit admissions. Approximately a quarter of the centers are conducting trials on NITS and, three quarters of the respondent suppose that the number of these procedures will increase in the future.

**Conclusions:**

There is a growing interest in Italy for NITS and this survey provides a clear view of the national management framework of these procedures.

**Supplementary Information:**

The online version contains supplementary material available at 10.1186/s12871-021-01514-3.

## Background

To reduce anesthetic and surgical techniques’ invasiveness, increasing their effectiveness and safety is the aim of new advances in thoracic surgery. Video-Assisted Thoracoscopic Surgery (VATS) is a minimally invasive surgical technique employed to diagnose and treat a variety of chest pathological conditions. Recently, its use and the increased use of the uniportal approach, promoted the possibility of avoiding general anesthesia (GA) and orotracheal intubation (OTI). Consequently, many thoracic surgical procedures such as Robot-Assisted Thoracoscopic Surgery (RATS) [1] and VATS lobectomy [2], VATS metastasectomy, segmentectomy [3], pneumothorax surgery [4], interstitial lung disease biopsies [5], endoscopic thymectomy [6] and minimally invasive esophagectomy [7] have been pursued without GA, by using locoregional techniques.

Although the intubated thoracoscopic surgery provides some advantages such as a guaranteed airway, a quiet and secure operating field, and a precise fraction of inspired oxygen, however, it is associated with intubation-related complications, such as residual neuromuscular blockade, and multiple effects on the respiratory system such as OTI related damages, and ventilation-perfusion mismatch [8].

Non-intubated technique in VATS procedures allows conducting surgeries through the use of regional anesthesia without GA, thus maintaining spontaneous breathing. During Non-Intubated Thoracic Surgery (NITS), iatrogenic pneumothorax provides lung isolation without needing a double-lumen tube or bronchial blocker.

Among the potential advantages of NITS are a lower incidence of intubation- and ventilation-related injuries [9], avoiding muscle relaxants and their potiental residual effects, reducing opioid use thus decreasing postoperative nausea and vomiting [9], other drug-related complications ^10,^ and lower operative morbidity [10].

In addition, it is known that intubated thoracic surgery results in a high risk of ventilator dependence, and difficult weaning in patients with myopathy and severe COPD with low FEV1 [11]. In contrast to this, NITS offers an option to avoid pathological lung damage by preserving negative inspiratory pressure instead of positive inspiratory pressure.

According Len at al., patients undergoing NITS compared to intubated patients had a faster recovery time in PACU, without significant differences in intraoperative oxygenation, despite a higher incidence of atelectases, pleural effusion, or pulmonary exudation [12]. However, the atelectases during NITS, is still debated extensively in the literature. More encouraging results are reported in the scientific literature showing that NITS could reduce postoperative complications, shorten hospital stays, and decrease the perioperative mortality rate. Thus, it appears to be safe, effective, and feasible for thoracic diseases [13].

Prisciandaro et al. assert that the NITS lobectomies for lung cancer are as effective and safe, especially concerning short terms outcomes, as intubated lobectomies [14].

Recently, a technique called SV-VATS tubeless has also been adopted, which provides in addition to NITS, avoidance of urinary catheter, central venous lines, and early removal of the chest tube. This technique is associated with reduced pain and shorter hospital stay [15, 16] .

Since we lack any recent data on this topic, we aimed to detect how widespread the use of this technique is in Italy, focusing on the centers that currently practice NITS on lung parenchyma.

## Methods

The present is a survey conducted in line with Checklist for Reporting Results of Internet E-Surveys (CHERRIES) checklist [17].

In September 2018 a multidisciplinary group (INFINITY- Italian Network For Investigation of Non-intubated Thoracic surgery) was assembled to study and evaluate the NITS experience in Italy. Thoracic surgeons and anesthesiologists from reference centers (defined as more than 100 operations per year), were involved.

The ethics committee of “Comitato Etico Interaziendale A.O.U. Città della Salute e della Scienza di Torino - A.O. Ordine Mauriziano - A.S.L. Città di Torino” considered that the submission of this survey was not necessary because it involves only professionals from various institutions in Italy and does not contain data involving individual patients, but data related to the daily clinical practice of individual professionals.

A web-based online questionnaire was designed and submitted to Italian centers by the INFINITY group in order to study the use of NITS. An initial draft was drawn up among all members of the INFINITY group, and the final version was approved after discussion until unanimous consensus was reached.

The questionnaire was sent on 26 June 2019 and participants were asked to reply by 31 November 2019.

The final questionnaire was made up of 32 questions focusing on surgical, anesthesiologic, and general issues regarding NITS and divided into 3 sections (Additional file 1: Appendix A). Section 1 mainly investigated the centers’ characteristics and previous NITS expertise; section 2 included questions addressed to surgeons and anesthesiologists performing NITS for pulmonary parenchymal resections, section 3 encompassed queries targeted at all the survey participants.

Survey recipients were asked to answer section 2 questions (i.e questions 7 to 23) considering only awake operations on the lung parenchyma (parenchymal NITS).

The survey was sent to a total of 58 General Thoracic Surgery units (Additional file 1: Appendix B). For each Institution, a thoracic surgeon and an anesthesiologist were required to answer according to the center’s experience.

## Results

After the data collection, the analysis of the results was performed. Afterward a database was created using Microsoft Excel software (v 2011, Office 365), it was divided according to the specialty and previous NITS technique experience. Inconsistent and implausible answers were excluded. Replies submitted by centers that partially completed the survey have been included. For each topic of the questions, the answers of surgeons and/or anesthesiologists with/without previous experience in NITS were then matched.

A descriptive analysis of the data obtained through Microsoft Excel software was carried out. Results were reported by numbers, percentage, average, median, interval range (IR), and interquartile range. A total of 50 surgeons and 48 anesthesiologists replied to the survey, achieving participation and feedback from 55 of 58 centers involved (95%).

### Centers characteristics and previous experience of NITS (Q1-Q6)

A median of 400 (IQR 300; 525) thoracic surgical operations per center per year were reported; a mean of 6 (IR 1;19) anesthesiologists for each center per team provided intraoperative management of these operations. Approximately one-fifth of centers (22%) found low confidence in performing regional anesthesia techniques as a major hurdle to performing NITS ((N 10, 83%),or because they did not consider the procedure safe (N 5, 42%), or the center’s type of surgery was not considered suitable for NITS (N 5, 42%).

The most common indications for NITS were pleural effusions (N 37, 86%), and pleural pathologies (N 35, 81%), followed by lymph node biopsies (N 21, 49%), interstitial disease (N 18, 42%), neoplasms (N 9, 21%), pneumothorax (N 8, 19%), and other pathologies (N 2, 5%). (Table 1).

**Table 1:** Awake surgeries

Q 5. For which pathologies do you perform non-intubated thoracic surgery procedures		
	%	N
Neoplasms	21	9
Interstitial disease	42	18
Pleural pathologies	81	35
Pleural effusion	86	37
Lymphnode biopsies	49	21
Pneumothorax	19	8
Other pathologies	5	2

**Fig. 1:**
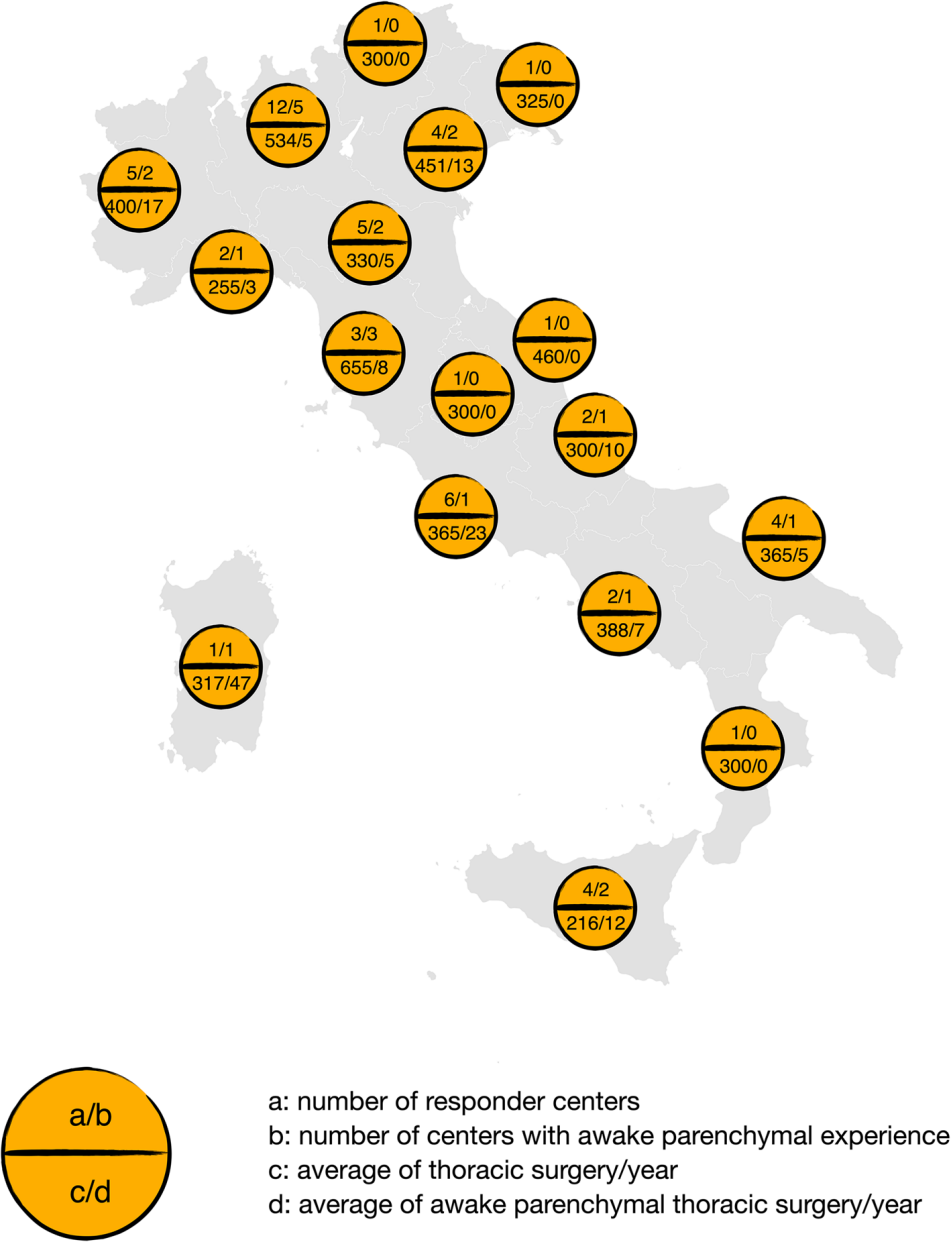
Data distribution in Italy

**Fig. 2:**
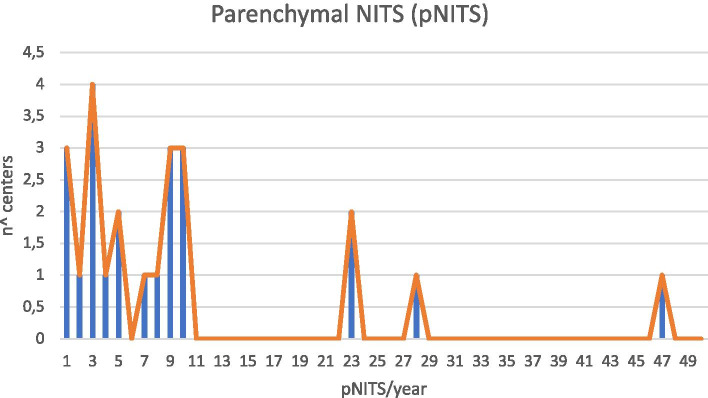
parenchymal NITS (parenchymal Non-intubated Thoracic Surgery)/Center

NITS on the lung parenchyma were performed in 21 (38%) centers, with a median of 8,25 (IQR 3,2; 10) surgical procedures per year. (Figs. 1 and 2).

### Indications and contraindications to parenchymal NITS (Q7-Q11)

In centers with previous experience on NITS, the survey shows that it may be performed in patients with severe comorbidity (N 28, 67%) or poor respiratory performance (N 28, 67%).

Only a minority of centers carried out NITS in patients without comorbidity (N 16, 38%) or good respiratory performance (N 6, 14%).

According to surgeons and anesthesiologists’ experience, obesity (N 26, 60%), preoperative NIV Non-Invasive Ventilation (NIV) and/or oxygen therapy (N 18, 42%), and major lung resections (N 21, 49%) prevailed among the possible contraindications to NITS (Table 2).

**Table 2:** Parenchymal Non-intubated Thoracic Surgery contraindications according centers with experience

**Q 8. Do you consider obesity a contraindication to NITS?**				
	**Anesthesiologists with experience**		**Surgeons with experience**	
	%	N	%	N
No	30	6	48	11
Yes	70	14	52	12
**Q 11. Do you think that NITS is contraindicated in major lung resections (lobectomy, anatomical segmentectomies)?**				
**Anesthesiologists with experience**				
	%	N	%	N
No	60	12	43	10
Yes	40	8	57	13

70% (N 14) of anesthesiologists considered an expected difficult airway a contraindication to NITS.

### Preoperative management of parenchymal NITS (Q12-Q12B)

90% (N 38) of the candidates underwent preoperative counseling for NITS, this was performed by anesthesiologists (N 37, 97%), thoracic surgeons (N 31, 82%), or pulmonologists (N 8, 21%). In 79% (N 30) of centers, the counseling was conducted by a multidisciplinary team.

### Intraoperative management of parenchymal NITS (Q13-Q23 AND Q28-Q29)

Aerosol with lidocaine was the most commonly used strategy to blunt cough reflex (N 22, 52%); other strategies used were vagal block (N 7, 17%), opioid, or intravenous lidocaine use, pleural nebulization of lidocaine (N 6, 14%).

A wide variety of regional anesthesia techniques has been employed during the perioperative period because different blocks provide intercostal nerve interception at different levels. Therefore, the use of paravertebral block (N 7, 37%), serratus anterior plane block (SAPB) (N 6, 32%), epidural catheter (N 6, 32%), intercostal nerve block (INB) (N 6, 32%), erector spinae plane block (ESPB) (N 4, 21%), pectoral nerve (PECS) 1 or 2 blocks (N 3, 16%), and subarachnoid anesthesia (N 1, 5%) was evaluated. Local anesthesia was added in about half of the cases (N 8, 42%).

In most cases, anesthesiologists performed intraoperative sedation with propofol (N 16, 84%), and 95% administered opioids (N 18), mostly remifentanil (N 13, 69%) (Table 3).

**Table 3:** Parenchymal Non-Intubated Thoracic Surgery intraoperative management according centers with experience

**Q 15. Locoregional anesthesia technique for intraoperative management**		
Anesthesiologists with experience	%	N
Local anesthesia	42	8
Only local anesthesia	0	0
Serratus Anterior Plane Block	32	6
Epidural catheter	32	6
Intercostal block	32	6
Pectoralis Nerve blocks	16	3
Erector Spinae block	21	4
Paravertebral block	37	7
Subarachnoid anesthesia	5	1
**Q 16. Intraoperative sedation**		
Anesthesiologists with experience	%	N
Propofol	84	16
Dexmedetomidine	5	1
Ketamine	5	1
Benzodiazepine	16	3
**Q 17. Intraoperative intravenous analgesia**		
Anesthesiologists with experience	%	N
Yes	95	18
Fentanyl	26	5
Sufentanil	0	0
Remifentanil	69	13
No	5	1

During NITS all patients were monitored according to standards with electrocardiogram and pulse oximetry. Devices monitoring anesthesia’s depth 21% (N 4), capnometry 79% (N 15), and invasive blood pressure 58% (N 11) were also added by anesthesiologists. In most cases oxygenation was improved through nasal cannulae (N 6, 32%), Venturi mask (N 7, 37%), and reservoir mask (N 4, 21%). Supraglottic devices were used by 10% (N 2) of anesthesiologists. In the case of conversion to open procedure, GA was often considered necessary (N 33, 79%).

One-third of respondents reported NITS to GA conversion at least once (N 13, 31%) which was done stopping the operation and turning the patient in a supine position by inducing GA and OTI (N 20, 49%), positioning laryngeal mask maintaining the lateral decubitus (N 17, 42%), proceeding to lateral decubitus intubation with video laryngoscope (N 2, 5%), or ventilating the patient with a face mask while keeping the lateral decubitus (N 1, 2%).

Generally, the medical staff in centers that performed NITS operations involved the most experienced surgeons and anesthesiologists of the center (N 31, 74%). Thoracic surgeons preferred an uniportal approach in the procedure (N 13, 57%), less frequently a two-port (N 5, 22%) or three-port (N 1, 4%) approach was used.

### Advantages and risks of NITS (Q24-Q27)

Evaluation of potential advantages and risks has been conducted in all centers, even with no previous NITS experience.

The survey results in the evaluation of the advantages have reported in about half of the cases a reduction in postoperative Intensive Care Unit (ICU) admission (N 39, 52%), a faster recovery (N 62, 80%), a lower incidence anesthesia-related complications (N 44, 57%), a lower incidence of damage induced by mechanical ventilation (N 47, 61%), less stress for the patient (N 33, 43%), and lower costs (N 16, 21%).

More than half of VATS group centers showed a lower degree of atelectasis in the postoperative phase with the NITS than with GA (N 48, 64%) (Supplementary Table S1).

Respondents identified as potential risk of NITS a possible difficulty in airway management (N 63, 74%), hemodynamic instability (N 17, 20%), a poor patient cooperation (N 46, 54%), cough and patient movements during surgery (N 65, 76%), possible increase in surgical timing (N 19, 22%), and the management of any intraoperative complications (N 58, 68%) (Table 4).

**Table 4:** Potential non-intubated thoracic surgery risks

Q 27. Potential Non-intubated Thoracic Surgery risks according to centers’ experience				
	Centers with experience		Centers without experience	
	%	N	%	N
Airway management	79	33	70	30
Hemodynamic instability	21	9	19	8
Poor patient cooperation	50	21	58	25
Cough and patient movements during surgery	81	34	72	31
Possible increase in surgical times	26	11	19	8
Management of any intraoperative complications	71	30	65	28

### Future perspectives (Q30-Q32)

In 22% of centers (N 19), NITS studies were underway or undergoing approval. Respondents in most cases believed in a proliferation of these procedures in the future (N 60, 72%), and were willing to visit other centers or share information about NITS management (N 85, 95%).

## Discussion

This is the first survey investigating NITS’ diffusion in Italy. Fifty-eight thoracic surgery centers were involved and a response rate of 86% surgeons and 83% anesthesiologists was obtained. Considering the number of centers reached and the high response rate, the reported data illustrates the real-life conditions and spread of awake thoracic surgery in Italy.

According to the data collected, NITS is used for parenchymal operations in 38% of cases (parenchymal NITS), such as lung atypical resections. Fifty-eight percent of respondents consider a major lung resection a contraindication to parenchymal NITS, therefore in Italy, unlike other countries, neither lobectomies nor pneumonectomies are regularly performed using parenchymal NITS.

From our data, parenchymal NITS was mainly performed on patients with severe comorbidities or impaired respiratory function, confirming the literature findings [18, 19].

The growing interest in this technique is confirmed by the numerous ongoing studies and by the fact that the majority of respondents thinks that NITS operations will increase in the coming years. For this reason, almost all of the participants are interested in visiting centers where NITS is practiced to master it.

Seventy-eight percent of respondents performs NITS procedures; the primary reason for not performing NITS surgery was a lack of confidence in using regional anesthesia techniques 83%). This could be due to the necessity of having a good performance of the locoregional technique, which is essential for the operation’s success.

Regarding intraoperative management, the anesthesiologists used heterogeneous techniques, also descripted in literature such as thoracic epidural analgesia [13, 20], ESPB, PVB, or INB [21].

Unlike a previous European survey, we did not observe a prevalence of INB [22]. In this survey, insufficient analgesia was observed in 42% of cases, where the infiltration of local anesthetic at the level of surgical wound was deemed necessary. To date, there is no standardization in locoregional procedures for parenchymal NITS, the variability seems to depend on the center’s and operator’s expertise.

According to most authors patients have to be sedated with intravenous propofol to maintain a Ramsay sedation score between III and IV, and opioids can be given to maintain a normal respiratory rate [14]. During the procedure, patients are usually placed in the lateral decubitus position according to surgical needs.

To improve the patient’s comfort during the procedure, anesthesiologists prefer sedoanalgesia with Propofol (84%) or Remifentanil (68%) rather than benzodiazepines (16%), combined with locoregional anesthesia.

Cough reflex control is necessary for surgical procedure safety, in 52% of cases aerosolized lidocaine was used due to the simplicity in execution compared to vagal nerve block, a technique described by Huang and Zhang [2, 11].

This survey shows that a predicted difficult airway is considered to be a contraindication to NITS more for anesthesiologists who are not performing NITS than those who are (83% vs 70%). Obesity is considered a contraindication to parenchymal NITS more for anesthesiologists than for surgeons (70% vs 52%), probably for a predicted difficult airway, although there is no unambiguous literature evidence on this issue [23, 24]. Regarding preoperative NIV, our results are opposed to a study by Kiss et al. whereby chronic preoperative NIV as well as severe myopathy and long-standing pulmonary fibrosis were inclusion criteria for NITS and exclusion criteria for GA. Even on this topic there is heterogeneity at the NITS indication [25]. Although effective in treating intraoperative hypoxia and hypercapnia, NIV is not routinely used in most of our centers to avoid applying positive pressure to the operated lung.

One could hypothesize that contraindications to parenchymal NITS execution are inversely proportional to the center’s experience.

Concerning airway management, this survey confirms that the use of nasal cannulas, Venturi masks, or reservoir masks ensure adequate oxygenation during parenchymal NITS surgery, as reported by Huang and Bedetti [19, 26].

The use of the laryngeal mask (LMA), described in other studies [26, 27], has been reported only in 10% of cases to maintain spontaneous breathing and to avoid the risks associated with OTI and mechanical ventilation. LMA requires a deeper anesthetic plan to allow its tolerance, it is used by 42% of anesthesiologists.

In the literature, the conversion rate is 2% to OTI and 0.2% to thoracotomy [19]. Among the data we collected, one-third of the anesthesiologists experienced a conversion to GA at least once. During the conversion 49% of the respondents interrupted the surgery and positioned the patient supine to proceed to GA and OTI, 42% positioned a LMA in lateral decubitus, 5% of the respondents maintained the patient in lateral decubitus by performing OTI with videolaryngoscopy (5%), 2% provided mask ventilation, and the remaining 2% used a different technique (namely Fastrack, AirTraq).

During parenchymal NITS, coughing and movements during the procedure (81%), and airway management (79%) are the most feared risks, according to our data. Several articles confirm our findings reporting intraoperative movements, refractory hypoxemia (oxygen saturation < 85% for more than 5 min), continuous cough despite aerosolized lidocaine, and uncontrolled bleeding as the main causes of conversion to GA [18, 28].

A study conducted by Mineo et al. indicated NITS as a safe technique to be adopted in particular contexts such as pleural effusions, mediastinal masses, and lung biopsies. Our survey aimed at providing a more updated, and a broader picture of the situation compared to Mineo et al. by including all Italian major reference centers 29.

As stated by Pompeo et al., most of the experienced centers in Italy (62%) consider parenchymal NITS a way to avoid ICU admissions in the postoperative period, in order to decrease costs [22]. NITS could also help decongest the ICU considering also the worldwide shortage of human resources. Other important advantages highlighted by our survey are a shorter postoperative hospitalization time (90%), and reduced mechanical ventilation stress (64%) especially in fragile patients. According to surgeons’ opinion parenchymal NITS (69%) has a smaller impact on the intraoperative atelectases development than GA, as pointed out by Furák et al. assuming that this was due to lower air leakage during NITS and an optimal parenchymal suture [30]. Contrariwise, 20% of respondents found that intraoperative atelectases were more frequent in parenchymal NITS than in GA. Atelectasis is a very controversial and debated issue; in fact, there are studies highlighting their increased incidence during NITS [12]. From the results of our survey, we suggest that perhaps the benefit on atelectasis provided during NITS can be obtained in patients undergoing non-parenchymal surgery, whereas when parenchymal surgery is considered, there is a higher incidence of atelectasis in NITS than in GA.

Recently, a consensus was also drawn up with all the most relevant technical points for safe execution of NITS, facilitating decision-making in this clinical setting [31]. With this survey we do not want to replace it, but we want to propose a national network of communication and help in an attempt to grow in expertise in a technique not yet widely performed nationwide.

In regards to future perspectives, there is a consistent and great interest in NITS and 72% of respondents believed that the use of NITS will increase in the future.

A full evaluation of the survey reveals less divergence of responses between surgeons and anesthesiologists in parenchymal NITS centers and more divergence between anesthesiologists in centers doing parenchymal NITS and in those not doing parenchymal NITS.

Moreover, the centers with the most experience found fewer contraindications and estimated the benefits greater than the risks.

The limitations of the study are mainly due to the intrinsic characteristics of surveys: there is a significant risk of detection bias for the subjectivity of the outcomes, even if expressed in numerical data. Moreover, despite widespread dissemination of the survey, not all centers in Italy were reached.

The main strengths include the high response rate obtained, considering the specificity of the subject [18], and its novelty value since it represents the first survey involving Italian thoracic surgery reference centers.

## Conclusions

Despite the fact that NITS procedures are frequently performed in reference centers in Italy, parenchymal NITS are seldom performed. Since surgical techniques are evolving more and more towards minimally invasive approaches, we believe that a similar trajectory should be followed by anesthesiologists as well when performing awake procedures. However, given the growing interest shown by the survey participants, we can suggest that parenchymal NITS is becoming increasingly popular in Italy, also due to the various advantages it offers.

Many centers are preparing or performing scientific studies, and centers that do not practice NITS have a strong interest in implementing this technique. Despite the intrinsic limitations of the study, we believe that this work can provide an outline of the current situation and promote the use of this technique and the spread of multicentric studies on its safety and effectiveness.

## Supplementary Information

**Additional file 1.**

## Data Availability

Survey data and data on participating centers are available as supplemental materials.

## Abbreviations

NITS: Non-Intubated Thoracic Surgery
VATS: Video-Assisted Thoracoscopic Surgery
GA: General anesthesia
OTI: Orotracheal intubation
RATS: Robot-Assisted Thoracoscopic Surgery
