# Supplementary material for: Italian survey on non-intubated thoracic surgery: results from INFINITY group
Source: BMC Anesthesiol. 2022 Jan 3;22:2. doi: 10.1186/s12871-021-01514-3 (PMC8722187; doi:10.1186/s12871-021-01514-3)
Supplement: Supplementary file 1 — Additional file 1. [file 12871_2021_1514_MOESM1_ESM.docx]

**SUPPLEMENTARY DATA**

**Appendix A**

Survey questions

*Section 1***-** *Centers characteristics and previous experience with NITS*

1. In your center, how many thoracic surgery operations do you perform on average each year?
2. In your center, how many anesthesiologists are involved in the intraoperative management of thoracic surgery patients?
3. In your center, do you carry out thoracic surgery in patients who are NOT under general anesthesia (NITS)?
4. If the answer to the question above is yes, on average how many thoracic surgery operations in patients NOT undergoing general anesthesia are conducted at your center in one year?
5. For which pathologies do you perform NITS procedures in your center?
6. On average, how many operations on the lung parenchyma in patients not undergoing general anesthesia are carried out at your center in one year?

*Section 2***-** *Indications and contraindications to NITS, Preoperative management, Preoperative management*

1. Which patients do you think could require "NITS” in your center?
2. Do you consider obesity a contraindication to "NITS"?
3. Do you consider an expected difficult airway a contraindication to "NITS"?

- 9b. How do you rate the difficulty of the airway?

1. Do you consider the use of preoperative NIV or oxygen therapy a contraindication to "NITS"?
2. Do you think that "NITS" is contraindicated in major lung resections (lobectomy, anatomical segmentectomies)?
3. Do you perform preoperative counseling in "NITS" candidates?

- 12b. If the answer to the question above is yes, which specialist carries it out?

1. In your center, do you have a formal path shared between anesthesiologists, thoracic surgeons and nurses for the management of patients undergoing "NITS" procedures?
2. Which strategy do you use in the first instance to reduce patient’s cough response during the operation?
3. Which regional anesthesia technique do you use for intraoperative management?
4. Do you perform intraoperative sedation?
5. Do you perform intravenous analgesia during the intraoperative period?
6. What additional monitoring do you use?
7. Have you ever converted the operation from "NITS" to general anesthesia?
8. In case of conversion of the thoracoscopic to thoracotomic procedure, do you consider the use of general anesthesia mandatory?
9. How do you manage the airway in patients undergoing "NITS" procedures?
10. Do you manage the airway differently based on the planned surgery?
11. How do you plan to manage any conversion to general anesthesia?

*Section 3* **-** *Advantages and risks of NITS, Future perspectives*

1. Compared to patients under general anesthesia, how often do patients undergoing NITS require postoperative intensive care unit admission (on equal terms)?
2. What do you think could be the added value of "NITS"?
3. How do you think lung atelectases occur during "NITS"?
4. What do you consider to be the risks of "NITS"?
5. Regarding your expertise area, are "NITS" procedures in your center preferably assigned to specialists with greater competence / experience?
6. In "NITS" operations performed with minimally invasive technique, which approach is preferably used in your center?
7. Regarding the "NITS" approach, are there active protocols or scientific studies in progress in your center?
8. How do you think the trend of NITS procedures in your center will change in the future?
9. Are you willing to visit other centers and host colleagues in your center to compare information about NITS?

**Appendix B**

*INFINTY group centers and corresponding members*

*Thoracic surgery Unit and Anesthesia and Intensive Care Unit*

1. SS. Antonio e Biagio e Cesare Arrigo Hospital, Alessandria

*Martuscelli L., Mancuso M.*

1. Ospedali riuniti Hospital, Ancona

*Gentili P., Majed R.*

1. Bolzano Hospital

*Fabbro L., Zaraca F.*

1. Cardarelli Hospital, Napoli

*Sellini M.*

1. Città della salute e della scienza Hospital, Torino

*Ruffini E., Lyberis P.*

1. Pugliese Ciaccio Hospital, Catanzaro

*Torchia F., Tuoro A.*

1. Arcispedale Sant’Anna Hospital, Ferrara

*Ragazzi R., Maniscalco P.*

1. Vito Fazzi Hospital, Lecce

*Micella A., Lopez C.*

1. Ospedale dell’angelo Venezia-Mestre

*Grassetto A., Lo Giudice F.*

1. Niguarda Hospital, Milano

*De Caria D., Rinaldo A.*

1. Maggiore Hospital, Novara

*Rena O., Cecci GP*

1. Padova Hospital

*Di Gregorio G., Nicotra S.*

1. Santa Croce e Carle Hospital, Cuneo

*Degiovanni C., Mazza F.*

1. San Camillo Forlanini Hospital, Roma

*Tritapepe L., Tessitore L.*

1. San Paolo Hospital, Milano

*Mistraletti G., Raveglia F.*

1. Sant’Andrea Hospital, Roma

*Massullo D., Andreetti C.*

1. Giuseppe Mazzini Hospital, Teramo

*Minora S., De Vico A.*

1. Santa Maria della Misericordia Hospital, Udine

*Copetti E., Morelli A.*

1. Santa Maria della Misericordia Hospital, Perugia

*Vinci D.*

1. Siena Hospital

*Marchetti L., Paladini P.*

1. Maggiore Hospital, Parma

*Tosi M., Ampollini L.*

1. Pisa Hospital

*Lucchi M.*

1. Verona Hospital

*Benato C.*

1. Careggi Hospital, Firenze

*Terrosi L., Gonfiotti A.*

1. Mantova e Cremona Hospital

*Castelli GP, Droghetti A.*

1. Riuniti Hospitals, Foggia

*Mariano K., Ardò N.*

1. Bellaria Hospital, Bologna

*Barbera N., Dolci G.*

1. Humanitas Hospital, Milano

*Difrancesco O., Novellis P.*

1. Humanitas Hospital, Catania

*Ciraolo R., Macrì P.*

1. Humanitas Gavazzeni Hospital, Bergamo

*Solinas C., Rizzardi G.*

1. Ca’ Granda Hospital, Milano

*Pesenti A., Tosi D.*

1. Istituto Nazionale Tumori (INT) Hospital, Milano

*Pardolesi A.*

1. San Martino Hospital, Genova

*Gratarola A., Pariscenti G.*

1. Istituto Mediterraneo per i Trapianti e Terapia ad Alta Specializzazione (ISMETT) Hospital, Palermo

*Arcadipane A.*

1. Istituto Europeo Oncologico (IEO) Hospital, Milano

*Antonaci A., Bertolaccini L.*

1. Istituto Nazionale Tumori Regina Elena (IFO) Hospital, Roma

*Coccia C., Mercadante E.*

1. Villa Sofia Hospital, Palermo

*Agneta G.*

1. Vittorio Emanuele Hospital, Catania

*Migliore M.*

1. Businco Hospital, Cagliari

*Carta G., Ferrari P.*

1. Sacro Cuore Hospital, Negrar (VR)

*Zamperini M., Viti A.*

1. San Gerardo Hospital, Monza

*Coppo A., Pirondini E.*

1. Ss. Annunziata Hospital, Taranto

*Carrieri F., Leggieri R.*

1. Casa Sollievo della Sofferenza Hospital, San Giovanni Rotondo (FG)

*Cavaliere L., Taurchini M.*

1. Monaldi Hospital, Napoli

*Rispoli M., Amore D.*

1. Di Circolo Hospital, Varese

*Cantone G., Rotolo N.*

1. San Giovanni Bosco Hospital, Torino

*Costa A., Fontana D.*

1. San Raffaele Hospital, Milano

*Magrin S., Bandiera A.*

1. Villa Scassi Hospital, Genova

*Cecchini A.*

1. Modena Hospital

*Stefani A.*

1. Gemelli Hospital, Roma

*Punzo G.*

1. Umberto I Hospital, Roma

*Bruno K., Anile M.*

1. Campus Bio Medico Hospital, Roma

*Martuscelli M., Crucitti P.*

1. Civili Hospital, Brescia

*Cattaneo S., Benvenuti M.R.*

1. AUSL Romagna Hospitals, Forlì and Ravenna

*Corso R., Davoli F.*

1. Ss. Annunziata Hospital, Chieti

*Camplese P.*

**SUPPLEMENTARY TABLES**

**TABLE S1**

|  | Tab S1. *Non-intubated Thoracic Surgery advantages*  Tab 4. *NITS advantages*  Q 24. Compared to patients General Anesthesia, those underwent Non-Intubated Thoracic Surgery required postoperative Intensive Care Unit admission: | | | | | | | | | | | | | | | | | | | | | | | | | | | | | | |  | | | |
| --- | --- | --- | --- | --- | --- | --- | --- | --- | --- | --- | --- | --- | --- | --- | --- | --- | --- | --- | --- | --- | --- | --- | --- | --- | --- | --- | --- | --- | --- | --- | --- | --- | --- | --- | --- |
|  | | |  | | Anesthesiologists with experience | | | | | | Surgeons with experience | | | | | | | | | | Anesthesiologists without experience | | | | | | Surgeons without experience | | | | | | | | |
|  | | |  | | % | | | N | | | | % | | | | | N | | | | | % | | N | | | | | % | | | | | N | |
| Difference cannot be assessed | | |  | | 16 | | | 3 | | | | 26 | | | | | 6 | | | | | 46 | | 6 | | | | | 55 | | | | | 11 | |
| The same percentage of cases | | |  | | 16 | | | 3 | | | | 17 | | | | | 4 | | | | | 8 | | 1 | | | | | 10 | | | | | 2 | |
| Less frequently | | |  | | 68 | | | 13 | | | | 57 | | | | | 13 | | | | | 46 | | 6 | | | | | 35 | | | | | 7 | |
|  | *Q 25. NITS advantages* | | | | | | | | | | | | | | |  | | | | | | | | | | | | | | | | | | | |
|  |  | | | *Anesthesiologists with NITS experience* | | | | | | *Surgeons with NITS experience* | | | | | | | | | *Anesthesiologists without NITS experience* | | | | | | | *Surgeons without NITS experience* | | | | | | | | | |
|  | |  | *%* | | | | *N* | | | *%* | | | | | *N* | | | | | *%* | | | *N* | | | *%* | | | | | *N* | | | | |
| Lower incidence of related anesthesia complications | | | 63 | | | | 12 | | | 57 | | | | | 13 | | | | | 43 | | | 6 | | | 62 | | | | | 13 | | | | |
| Lower incidence of damage induced by mechanical ventilation | | | 63 | | | | 12 | | | 65 | | | | | 15 | | | | | 50 | | | 7 | | | 62 | | | | | 13 | | | | |
| Faster patients’ recovery | | | 89 | | | 17 | | | | 91 | | | | | 21 | | | | | 57 | | | 8 | | | 76 | | | | | 16 | | | | |
| Lower access in Intensive Care Unit during the postoperative period | | | 47 | | | | 9 | | | 35 | | | | | 8 | | | | | 36 | | | 5 | | | 43 | | | | | 9 | | | | |
| Less stress for the patient | | | 42 | | | | 8 | | | 48 | | | | | 11 | | | | | 21 | | | 3 | | | 52 | | | | | 11 | | | | |
| Lower costs | |  | 16 | | | | 3 | | | 22 | | | | | 5 | | | | | 21 | | | 3 | | | 24 | | | | | 5 | | | | |
| Other | | | 0 | | | | 0 | | | 0 | | | | | 0 | | | | | 0 | | | 0 | | | 5 | | | | | 1 | | | | |
|  | Q 26. You believe that pulmonary atelectases that occurs during NITS are: | | | | | | | | | | | | | | | | | | | | | | | | | | | | | | | |  | | |
|  | | |  | | Anesthesiologists with NITS experience | | | | | | | | Surgeons with NITS experience | | | | | | | | Anesthesiologists without NITS experience | | | | | | | Surgeons without NITS experience | | | | | | | |
|  | | |  | | % | | | | N | | | | | % | | | | N | | | | % | | | N | | | | | % | | | | | N |
| Less than during general anesthesia | | |  | | 28 | | | | 5 | | | | | 69 | | | | 16 | | | | 64 | | | 9 | | | | | 90 | | | | | 18 |
| Greater than during general anesthesia | | |  | | 33 | | | | 6 | | | | | 9 | | | | 2 | | | | 14 | | | 2 | | | | | 10 | | | | | 2 |
| The same as during general anesthesia | | |  | | 33 | | | | 6 | | | | | 22 | | | | 5 | | | | 22 | | | 3 | | | | | 0 | | | | | 0 |
| It depends on the underlying pathology | | |  | | 6 | | | | 1 | | | | | 0 | | | | 0 | | | | 0 | | | 0 | | | | | 0 | | | | | 0 |
